# Prevalence and factors associated with hypertension and obesity among civil servants in Kaduna, Kaduna State, June 2012

**DOI:** 10.11694/pamj.supp.2014.18.1.3260

**Published:** 2014-07-21

**Authors:** Abisola Monisola Oladimeji, Olufunmilayo Fawole, Patrick Nguku, Peter Nsubuga

**Affiliations:** 1 Nigeria Field Epidemiology and Laboratory Training Programme, Abuja, Nigeria; 2 Epidemiology and Medical Statistics Department, University of Ibadan, Ibadan, Nigeria; 3 Global Public Health Solutions, Decatur, Georgia, USA

**Keywords:** Hypertension, overweight, obesity, body mass index, civil servants, Kaduna

## Abstract

**Introduction:**

Non-communicable diseases (NCDs) are a leading cause of adult mortality globally, accounting for 63% of all deaths in 2008 with nearly 80% of those deaths occurring in developing countries. These NCDs which include hypertension and obesity alongside their complications accounted for 27% of all deaths in Nigeria, in 2008. We conducted a study among Kaduna State civil servants to determine the prevalence of hypertension, overweight/obesity and also to identify associated behavioural factors.

**Methods:**

A cross-sectional design, with multi-stage cluster sampling technique was used. A structured questionnaire was used in gathering data on socio-demographics, physical activity, dietary habit, tobacco, and alcohol consumption. Blood pressure, body weight and height were measured, and body mass index (BMI) calculated. Descriptive statistics and logistic regression were used in identifying associations between these behavioural factors and hypertension/overweight/obesity.

**Results:**

A total of 801 civil servants, mean age 43±9 years were interviewed, of which 62% were male. Prevalence of hypertension, overweight and obesity were 29%, 35% and 27% respectively. Physical inactivity was the most prevalent behavioural factor, 91%, followed by unhealthy diet 90%, and cigarette smoking 6%. Prevalence of overweight/obesity was higher among the senior cadre than the junior cadre (69% versus 54%, p<0.01). Increasing age was an independent predictor of hypertension. Female respondents were four times more likely to be overweight/obese than males (AOR=3.7, 95%CI 2.5-5.4).

**Conclusion:**

Hypertension and overweight/obesity with their behavioural risks are prevalent among civil servants in Kaduna. Age and gender-specific public health strategies to promote healthy- living in the workplace are being advocated for with concerned authorities.

## Introduction

Developing countries are currently undergoing an epidemiological transition that has been previously observed in developed countries. As a result of this, African populations now suffer under the dual burden of infectious diseases and emerging chronic diseases [1].

These chronic diseases otherwise known as non-communicable diseases (NCDs) are the leading cause of adult mortality globally; accounting for 36 million, 63% of all deaths in 2008. Nearly 80% of these deaths occurred in low- and middle-income countries, with the highest proportion of deaths among those aged <70 years [2]. In Nigeria, the major NCDs which contribute to increased mortality in adults are hypertension, diabetes, cancers, chronic respiratory disease, and obesity [3].

However, most NCDs have been strongly associated and causally linked with four particular behaviours namely: tobacco use, physical inactivity, unhealthy diet, and harmful use of alcohol. These behaviours lead to key metabolic/physiological changes like raised blood pressure, overweight/obesity, hyperglycemia, and hyperlipidemia. Raised blood pressure has the highest attributable risk percent for death, 13%. This is followed by tobacco use 9%, raised blood glucose 6%, physical inactivity 6%, and overweight with obesity 5% [4]. These risk factors have been influenced by urbanization and improved socio-economic circumstances [5]. In Nigeria, the increasing use of motorized transport and sedentary types of occupation such as office work, accompanied by high risk dietary and lifestyle behaviours have had their effects on the occurrence of NCDs [5, 6].

In 2008, NCDs accounted for 27% of all deaths in Nigeria, with 42% of these deaths respectively occurring in males and females <60 years old [7]. This age-group constitute the economic backbone i.e., workforce of any country. The burden of NCDs has been shown to have impact, not only on the quality of life of affected individuals and their families, but also on the country's socioeconomic structure. In 2005 alone, Nigeria was estimated to have lost 400 million United States dollars in national income from premature deaths due to heart disease, stroke, and diabetes and would have an estimated four-fold increase in income loss by 2015 [8, 9].

There is however evidence that the morbidity and mortality from NCDs can be reduced by eliminating these high-risk behaviours and this can be achieved by increasing awareness of these risk behaviours and by timely use of preventive health services. A three-pronged solution consisting of public health surveillance, primary prevention, and secondary prevention has been proposed by experts. However public health surveillance of risk behaviours has been prioritized over primary and secondary prevention [10].

The World Health Organization has also documented evidence that up to three quarters of these NCDs can be prevented by addressing their risk factors [8], yet they have not been adequately characterised in most developing countries that now have a large share of the burden of the disease. Workplace Health Promotion (WHP) programmes targeting physical inactivity and unhealthy dietary habits, have been documented as being effective by the WHO/World Economic Forum in improving health-related outcomes such as obesity, diabetes and cardiovascular disease risk factors [9].

This study was conducted to determine the prevalence of raised blood pressure and overweight including obesity among civil servants in Kaduna City. We examined behaviours such as physical inactivity, tobacco use, harmful alcohol consumption, and unhealthy dietary habit among the civil servants, determined associations between the behavioural factors and the occurrence of these non-communicable diseases and assist in policy formulation for interventions against these non-communicable diseases.

## Methods

**Study area:** The study was carried out in the capital city of the state, Kaduna. The state is one of the 36 states that make up Nigeria. The Kaduna Capital City is an urban centre, housing the headquarters of most of the government ministries/agencies within the state. The state has 22 ministries with a workforce of about 20,000 people.

**Study Design/Sampling technique:** A cross-sectional study was conducted among civil servants in 10 randomly selected ministries using a two-stage cluster sampling technique in order to produce a representative sample of the workforce in the city. All the departments within each of the selected ministries were incorporated with all consenting civil servants interviewed. All pregnant civil servants during the period of study were excluded.

**Sample size determination:** The Leslie and Kish formula was used in estimating the sample size for the cross-sectional survey

n = g * (Z^2^αpq)/d^2^

Where: n= Minimum sample size, Zα at 5% significant level put together = 1.96 p = prevalence of hypertension at 27.1%.

Using the highest documented prevalence of hypertension among paid workers in Nigeria [11] d = level of precision (5%), q = 1- p and g = 2 (design effect for a cluster sample)

n = 2 x (1.96^2^ x 0.27 x 0.73)/0.05^2^; n= 605.74

Adjusting for non-response rate of 10%: =nr/(r - 1)

Where n = calculated sample size and r = 10 =605.74 x 10/(10 - 1) = 673.05 Estimated Sample Size = 673

### Ethical consideration

Ethical approval was obtained from the Kaduna State Ministry of Health Ethical Review board. Informed consent was taken from respondents before administering questionnaire or anthropometry. Names and addresses were excluded to maintain the confidentiality of the respondents. Participants had brief one-to-one discussion on health implications of risk behaviours and measures to reduce the risk of NCDs. Persons with borderline high blood pressure (BP) (i.e., measurements of 140/90mmHg) were counselled on the need to see a physician for further review and follow up. Those with marked elevations ≥180/110mmHg [12],were referred to the nearest hospitals for urgent medical treatment and management.

### Data Collection Instruments and Procedures

The instruments used in carrying out the survey included interviewer-administered structured questionnaire (with open-ended and closed questions), weighing scale for measuring body weight in kilograms (kg), stadiometer for the measurement of height in metres (m) and sphygmomanometer for measurement of blood pressure in millimetres of mercury (mmHg).

The World Health Organization (WHO) STEPwise approach was employed, adopting the Centers for Disease Control and Prevention (CDC) Behavioural Risk Factor Surveillance System (BRFSS)/ Chronic Disease Indicators (CDI) [13, 14]. The WHO STEPwise approach to chronic disease risk factor surveillance (STEPS) focuses on obtaining core data on established risk factors that determine major disease burden. The instrument covers three different levels of “steps” of risk factor assessment i.e., questionnaire on socio-demographics, physical measurements and biochemical. The Chronic Disease Indicators (CDI) is a set of indicators developed to allow for uniform definition, collection, and reporting of chronic disease data that are important in public health. The CDI represent a wide spectrum of conditions and risk factors such as physical activity and nutrition, tobacco and alcohol use, cancers, cardiovascular disease, and diabetes.

For the purpose of the study, the first two WHO steps were employed. The first part of our study instrument collected data on respondents’ demographic, socioeconomic, behavioural and medical history. The second part took respondents’ physical body measurements (anthropometry). Fifteen research assistants were trained to administer the questionnaires and take anthropometric measurements. Training was done by the investigator over a period of 2 days. Performance of RA was assessed before they went to field to commence data collection.

Research assistants obtained physical measurements and administered a structured questionnaire. Height was measured to the nearest centimeters, using standard height meter rules, with the participants standing upright. Weight was measured using calibrated weighing scales and the participants lightly clothed. Blood pressure was measured on the upper left arm after at least 10 minutes of rest using a mercury sphygmomanometer.

### Pre-testing data collection instrument

The questionnaire was pre-tested on 50 civil servants within a ministry outside the sample for study. This was to identify ambiguity and likely duration of administering survey instruments.

### Data management and analysis

Data were entered, cleaned and edited for inconsistencies before analysing with SPSS version 19 and Epi info version 3.5.3. Questionnaires not meeting quality control checks were excluded from analysis. Descriptive and analytical statistics were used in summarizing the data. Descriptive statistics involved the use of frequencies, proportions and tables. Analytical statistics through bivariate analysis and multivariate logistic regression were performed to identify factors associated with the occurrence of hypertension, overweight and obesity. The chi square test was used in determining statistically significant associations while factors with p values <0.05 were included in the logistic regression model. Adjusted odds ratios (AORs) were determined with 95% confidence interval (CI) to identify independent factors.

### Operational Definitions

**Hypertension** was defined as a measured blood pressure ≥140 mmHg systolic and/or ≥90 mmHg diastolic or self-reported use of drug treatment for hypertension irrespective of measured blood pressure [15, 16].

**Body Mass Index BMI** was defined based on the WHO Classification [17]: Normal range BMI (healthy weight) = 18.5-24.9kg/m^2^; underweight <18.5kg/m^2^; overweight = 25.0-29.9kg/m^2^; obesity ≥0.0kg/m^2^.

**Unhealthy diet** was defined as absence of fresh fruits and cooked vegetables in the daily diet of the workers.

**Physical inactivity** was defined as absence of non-vigorous physical activity for at least 30 minutes ≥5 days of a week or vigorous physical activity for 20 minutes in ≥3 days of a week.

**Binge drinking** was defined in males as consumption of ≥5 drinks of alcohol at one sitting and ≥4 drinks in females at one sitting.

**Tobacco smoking** was defined based on civil servants reporting current smoking of cigarettes.

## Results

The mean age of the 801 respondents was 43 years standard deviation (SD) 9.0 years with 62% being male. Majority 80.0% of the civil servants were married. Two-thirds, 62% were Christians and 78% had a minimum of tertiary education. Seventy-two percent of the civil servants belonged to the senior staff cadre (salary grade level 8 and above) of the Nigerian salary scale which ranges from grade 1 to 17 (Table 1).

**Table 1:** Socio-demographic Characteristics of Civil Servants in Kaduna, Kaduna State 2012

Variable	% (95% CI)
**Sex**	
Male	62.2 (58.7-65.5)
Female	37.8 (34.5-41.3)
**Age (years)**	
20-29	9.7 (7.8-12.1)
30-39	21.1 (18.4-24.1)
40-49	42.4 (39.0-46.0)
50-59	24.7 (21.8-27.9)
≥ 60	2.0 (1.2-3.3)
**Marital Status**	
Single (never married)	13.7 (11.5-16.4)
Currently married	77.9 (74.8-80.7)
Separated	1.0 (0.5-2.0)
Divorced	0.6 (0.2-1.5)
Widowed	6.7 (5.1-8.8)
**Religion**	
Christianity	61.7 (58.2-65.0)
Islam	38.3 (35.0-41.8)
**Educational Status**	
No formal education	1.7 (1.0-3.0)
Arabic school	0.7 (0.3-1.7)
Primary education	6.6 (5.0-8.6)
Secondary education	13.2 (11.0-15.8)
Tertiary education	62.9 (59.5-66.3)
Post-graduate training	14.9 (12.4-17.4)
**Job cadre (Salary grades) n = 765**	
Junior (GL 1-7)	24.6 (21.6-27.8)
Senior staff (≥GL 8)	75.4 (72.2-78.4)

### Prevalence of the Behavioural Factors, Hypertension, Overweight and Obesity

Fifty-four percent of the civil servants reported being engaged in one form of physical activity or the other 30 days prior to the study. Of this proportion, 66% fell under the vigorous physical activities such as brisk walking, jogging, or football. Based on the CDI definition for physical activity, only 9% of the total respondents were physically active. Physical inactivity thus accounted for the most prevalent behavioural factor, 91%, closely followed by unhealthy diet, 90%. Seven percent of the civil servants reported smoking in the past, while 6% reported current cigarette smoking. Cigarette smoking was reported predominantly among the males. Binge drinking was reported among 2% of the civil servants.

The overall prevalence of hypertension among Kaduna State civil servants was 29%. Eleven percent of them was known hypertensive, and had been on anti-hypertension medications at least two weeks prior to the survey. Eighteen percent were newly discovered to have hypertension. Close to half of the civil servants, 47% had an immediate family member with history of hypertension. Hypertension was more prevalent among civil servants aged 40 years and above compared with those younger than 40 years (38% versus 9%, p<0.01). After stratifying by age, the prevalence of hypertension was higher among the female ≥ 40 years (41%), not currently married (41%), those that binge drink, and those with positive family history of hypertension (43%) (Table 2).

**Table 2:** Factors associated with Hypertension, Overweight and Obesity among Civil Servants in Kaduna, Kaduna State, 2012 [Hypertension (BP ≥140/90mmHg)]

	Hypertension		Overweight and Obese	
	< 40 years [%, 95% CI]	≥ 40 years [%, 95% CI]	< 40 years [%, 95% CI]	≥ 40 years [%, 95% CI]
**Sex**				
Male	6.9 (3.7-11.7)	36.9 (31.9-42.1)	36.4 (29.1-44.2)	60.6 (55.2-65.7)
Female	12.5 (6.8-20.7)	40.5 (34.1-47.1)	67.1 (56.6-76.4)	87.9 (83.0-91.7)
**Marital status**				
Currently married	9.2 (5.2-14.9)	37.9 (33.6-42.3)	50.0 (41.6-58.4)	70.5 (66.3-74.5)
Not currently married	8.5 (4.2-15.0)	40.8 (29.9-52.5)	43.7 (34.4-53.4)	77.1 (66.2-85.8)
**Educational status**				
Below tertiary	10.3 (4.3-20.3)	43.8 (35.2-52.7)	36.4 (24.5-49.6)	67.8 (59.0-75.8)
Tertiary and above	8.5 (5.1-13.1)	36.7 (32.3-41.3)	50.5 (43.3-57.7)	72.3 (67.9-76.5)
**Job cadre**				
Junior staff	7.1 (2.9-14.1)	39.8 (30.7-49.5)	41.5 (31.2-52.3)	65.3 (55.5-74.2)
Senior staff	10.1 (5.8-16.0)	37.7 (33.2-42.3)	53.7 (45.3-62.1)	73.3 (68.9-77.3)
**Diet**				
Unhealthy diet	8.2 (5.2-12.3)	38.5 (34.3-42.9)	48.0 (41.5-54.5)	70.7 (66.5-74.6)
Healthy diet	18.8 (5.0-43.0)	36.1 (24.8-48.6)	35.7 (14.4-62.4)	77.2 (65.0-86.7)
**Binge drinking**				
Yes	11.1 (0.6-43.9)	83.3 (40.9-99.2)	55.6 (24.0-84.0)	80.0 (33.4-99.0)
No	8.8 (5.7-13.0)	37.8 (33.8-41.9)	47.0 (40.6-53.4)	71.3 (67.3-75.0)
**Cigarette smoking**				
Current smokers	6.3 (0.3-27.2)	35.5 (20.3-53.3)	37.5 (18.4-61.5)	36.7 (21.8-54.6)
Non-smokers	9.0 (5.8-13.4)	38.6 (34.3-43.0)	45.5 (39.1-52.1)	74.6 (70.5-78.4)
**Physical activity**				
Physically inactive	8.8 (5.6-13.2)	38.3 (34.1-42.5)	48.3 (41.6-55.1)	71.9 (67.9-75.7)
Physically active	9.4 (2.4-23.4)	38.5 (24.3-54.3)	40.6 (24.8-58.1)	64.1 (48.3-77.9)
**Body Mass Index**				
Overweight & Obese	15.9 (10.0-23.6)	43.5 (38.6-48.5)	-	-
Healthy weight	3.2 (1.0-7.5)	26.5 (20.0-33.8)	-	-
**Family history of hypertension**				
Yes	10.7 (6.1-17.1)	43.5 (37.5-49.6)	-	-
No	7.2 (3.6-12.8)	33.9 (28.7-39.4)	-	-

**Figure 1:**
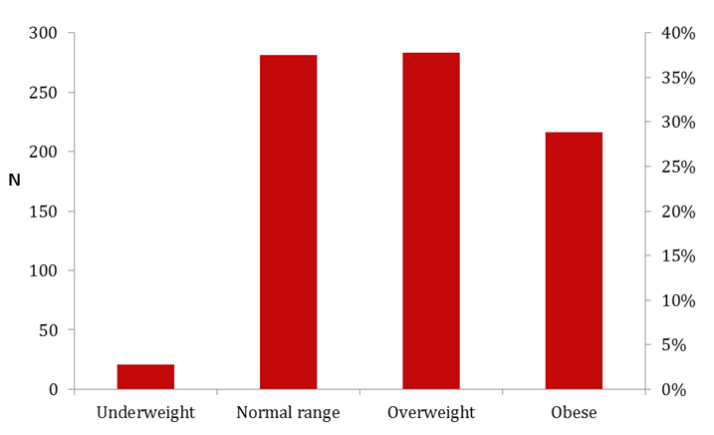
Body mass index (BMI) distribution of civil servants in Kaduna, Kaduna state, 2012

A third of the civil servants were overweight and another 27% were obese. While a small proportion, 2.6% were underweight, 35% had healthy weight (Figure 1).

Overall, 64% were either overweight or obese. The prevalence of overweight and obesity was higher among females aged ≥ 40 years compared with their male counterparts of the same age group (88% versus 61%). Irrespective of the age category a higher prevalence of overweight and obesity was found among civil servants with a minimum of tertiary education, physically inactive and those that binge drink (Table 2).

### Factors Associated with Hypertension and Overweight/Obesity

Cutting across the age categories, a higher percentage of hypertension, overweight and obesity was found among the female civil servants (Table 2). Higher proportion of physically inactive people civil servants were overweight and obese compared with their physically active counterparts irrespective of age category (Table 2).

A regression model consisting of the age of the civil servants which was categorized into five groups, their marital status, and family history of hypertension was built. On this model, civil servants aged ≥ 40 years were at significantly higher risk of hypertension compared with those < 40 years (AOR = 6.7, 95%CI 4.1-11.0), Table 3.

**Table 3:** Adjusted Odds Ratio of Predictors of Hypertension among Civil Servants in Kaduna, Kaduna State, 2012

	AOR	95% CI	p-value
**Age (in years)**			
≥ 40	6.7	4.1-11.0	<0.01 (55.8-71.0)
< 40	1	Ref.	
**Marital status**			
Not currently married	0.9	0.6-1.4	0.73 (41.6-58.4)
Currently married	1	Ref.	
**Family history of hypertension**			
Positive history	1.5	1.1-2.1	0.01 (41.9-75.2)
No family history	1	Ref.	

Civil servants with positive family history of hypertension were twice likely to be hypertensive compared with those without a positive family history (AOR 1.5, 95%CI 1.1-2.1), Table 3. There were no statistically significant associations between the behavioural factors and elevated blood pressure.

After adjusting for age, Kaduna female civil servants were found four times more likely to be overweight/obese than males (AOR = 3.7, 95%CI 2.5-5.4).

## Discussion

The prevalence of hypertension obtained from our study among Kaduna civil servants is similar to that documented among paid workers in Ilorin, Nigeria (27.1%) by Oghagbon EK, Okesina AB and Biliaminu SA in 2008 [11]. The prevalence obtained from these two studies are however lower than the estimated hypertension prevalence of 42.8% for the entire country (Nigeria) in same 2008 [8]. The higher prevalence at country level might have been due to involvement of the elderly and retirees in the study population. Our study found no statistically significant relationship between elevated blood pressure and the behavioural risk factors. However, a higher prevalence of hypertension was reported among the binge drinkers (40% vs. 29%). Previous studies have also shown correlations between harmful alcohol consumption and the occurrence of hypertension [18–20].

Compared with the estimated country prevalence of overweight and obesity in 2008, our study found a higher prevalence of overweight and obesity seen among the civil servants (35.3% vs. 26.8% and 27.0% vs. 6.5% respectively) [8]. This might be explained by the essentially sedentary lifestyle associated with this group of the population

Older people tend to have a higher BMI when compared to the younger population [21, 22]. The older age group occupy the senior cadre jobs and are essentially at the decision-making level, with less frequent physically demanding routines. Apart from the older age group, studies have shown the female sex to be at higher risk of overweight/obesity compared with males [23–25]. Our study found a 4-fold higher prevalence of overweight/ obesity among the female civil servants.

Increased risk of hypertension has been shown to rise with increasing age. This was demonstrated in our study with the older age groups having higher prevalence of elevated blood pressure compared with the younger ones. Although not statistically significant higher prevalence of hypertension were seen among the physically inactive and binge drinkers.

Overweight and obesity are documented risk factors for cardio-vascular disease; this was demonstrated in the two-fold risk of hypertension seen among the obese respondents.

Our study was conducted in order to determine the prevalence of hypertension, overweight and obesity among this at risk group of workers with essentially sedentary lifestyle, and identify associated behavioural factors. The study did not however establish causal relationship between these behavioural factors and selected NCD due to the cross- sectional study design employed. The behavioural factors elicited were respondents’ self-report, as such there might be exaggeration of socially acceptable practices from them. The clinical diagnosis of hypertension is made based on several blood pressure measurement readings while a single reading was used in our study. However, resting measurements were taken by trained personnel. Nevertheless, our study has shed light on the occurrence and current prevalence of hypertension, overweight/obesity and their associated factors in the various categories of civil servants in Kaduna, Kaduna State.

## Conclusion

Hypertension, overweight and obesity are prevalent among civil servants in Kaduna, Kaduna State. Physical inactivity and unhealthy dietary habits are the most prevalent behavioural factors. Age and BMI were independent predictors of hypertension; female sex was at higher risk of overweight/obesity. There is a need to introduce ways of increasing physical activity and healthy diet among the civil servants in Kaduna. The increased likelihood of hypertension among the older age-groups and obese, justifies the need for routine screening for hypertension among the civil servants especially the older age-groups, obese and those with positive family history of hypertension. Effective smoking cessation services and responsible alcohol-intake advocacy should be introduced into the work environment to assist cigarette smokers quit smoking and binge drinkers drink responsibly. Age and gender- specific public health strategies to promote healthy-living in the workplace are being advocated for with concerned authorities [21].
